# Allergy to sunflower seed and sunflower butter as proposed vehicle for sensitization

**DOI:** 10.1186/s13223-014-0065-6

**Published:** 2015-01-08

**Authors:** Elana Lavine, Moshe Ben-Shoshan

**Affiliations:** Department of Pediatrics, Pediatric Allergy Clinic, Humber River Regional Hospital, c / o 404-586 Eglinton Avenue East, Toronto, ON Canada M4P 1P2; Department of Pediatrics, Division of Pediatric Allergy and Clinical Immunology, McGill University Health Centre, Montreal, QC Canada

**Keywords:** Sunflower seed, Allergy, Risk factors

## Abstract

**Background:**

It is hypothesized that household exposure to allergenic proteins via an impaired skin barrier, such as atopic dermatitis, may contribute to the development of IgE sensitization. Household presence of peanut is a risk factor for the development of peanut allergy in children. Sunflower seed butter is a peanut-free alternative to peanut butter, and sunflower seed allergy is an uncommon but reported entity.

**Case presentation:**

A 3 year old boy presented with oral discomfort that developed almost immediately after he ate sunflower seeds for the first time. He was given a dose of diphenhydramine. Subsequently he vomited, and his symptoms gradually resolved. A similar episode occurred to a commercial snack made with sunflower seed butter. Skin prick testing demonstrated a large positive (10 mm wheal) wheal-and-flare response to a slurry of fresh sunflower seed within 3–4 minutes associated with severe pruritus.

This child has an older sibling with confirmed peanut allergy (PNA). After the PNA diagnosis was made, the family home became peanut-free. In lieu of peanut butter, sunflower butter was purchased and eaten frequently by family members, but not by the child reported herein.

Subsequent to the episodes above, the child ate a bread roll with visible poppy seeds and developed itchy throat, dyspnea, and urticaria. Epicutaneous skin testing elicited a >10 mm wheal size within 3–4 minutes in response to a slurry of whole poppy seeds and 8 mm to fresh pumpkin seed, which had never been consumed.

**Conclusions:**

A case of sunflower allergy in the context of household consumption of sunflower butter has not yet been reported. We suggest that homes which are intentionally peanut-safe may provide an environment whereby infants with impaired skin barrier are at increased risk of allergy to alternative “butter” products being used, via cutaneous exposure to these products preceding oral introduction to the child.

## Background

Given that studies report relatively rapid increase in worldwide prevalence of food allergy in the last decade [1], research has aimed to identify modifiable environmental factors that might contribute to this increase. However, the factors involved have not yet been established. While early ingestion of allergenic foods may promote the development of tolerance [2], one prominent hypothesis suggests that household exposure to allergenic proteins via an impaired skin barrier may contribute to the development of IgE sensitization to those food proteins. Both household presence of peanut [3] and development of atopic dermatitis in infancy [4] are risk factors for development of peanut allergy in children.

Seeds are now recognized as a significant food allergen with potential for anaphylaxis. Sesame and mustard are both priority food allergens on Health Canada’s list; they must be identified on product labels [5]. Although they are the most common seed allergies reported, reactions to other seeds can also be immediate and severe [6,7]. We report a case of sun flower seed allergy in the context of impaired skin barrier and frequent home exposure to sunflower seeds in the form of sunflower “butter”, one available substitute for peanut butter.

## Case presentation

A 3 year old boy presented with oral discomfort that developed almost immediately after he ate sunflower seeds for the first time. He was given a dose of diphenhydramine. Subsequently he vomited, and his symptoms gradually resolved.

Some weeks later he was given a snack bar made with sunflower seed “butter”, and after a small bite, vomited, complained of discomfort of his mouth and tongue, and seemed sleepier than usual. Parents monitored him at home and no medication was given. They sought an allergy consultation to assess for possibility of sunflower seed allergy.

This child has an older sibling with confirmed peanut allergy (PNA). After the PNA diagnosis was made, the family home became peanut-free. In lieu of peanut butter, sunflower butter was purchased and eaten frequently by family members, but not by the child reported herein. However, he had been consuming sunflower oil for several years without incident. He had also eaten whole sesame seeds and flaxseed. He had a history of mild infantile atopic dermatitis, which had improved over time.

Subsequent to the episodes above, the child ate a bread roll with visible poppy seeds and developed itchy throat and dyspnea. Parents administered an epinephrine auto-injector and brought him to the emergency department, where he developed facial urticaria. He was monitored and discharged home after an uneventful period of observation.

Epicutaneous skin (prick) testing demonstrated a large positive (10 mm wheal) wheal-and-flare response to a slurry of fresh sunflower seed within 3–4 minutes associated with severe pruritus. A diagnosis was made of sunflower seed allergy and recommendations given for strict avoidance and availability of an epinephrine auto-injector at all times. A similar reaction (>10 mm wheal size within 3–4 minutes) was observed in response to epicutaneous testing with a slurry of whole poppy seeds. A smaller reaction (8 mm wheal size) was observed to fresh pumpkin seed, which had never been consumed. Parents were counseled with regards to avoidance of all seeds, and declined skin testing with mustard seed.

## Discussion

### Allergenic properties of seeds

Sunflower and other seeds are small embryonic plants encased in seed coats. Common seeds include sesame, poppy, sunflower, mustard, flaxseed and canola (rapeseed). The 2S albumin storage proteins are major allergens found in the seeds of a wide range of mono and di-cotyledonous plants. They function as storage proteins, providing nutrients during germination and to the growing plant. 2S albumins, named as such for their sedimentation coefficient, are found in peanuts, certain tree nuts, and many seeds including that of sunflower (*Heliantus annuus*) [8].

While they demonstrate a high level of polymorphism, typical characteristics of 2S albumin storage proteins include compact structure, disulfide bonds which confer stability under conditions of heating or pH extremes, and glycosylation, conferring additional stability. These qualities allow for resistance to harsh conditions such as acidic pH and proteolytic digestive enzymes, allowing these allergens to reach the gastrointestinal immune system with intact allergenic epitopes that can elicit IgE responses [8].

### Sunflower seed, its products, and allergy

Although uncommon, sunflower seed allergy has been reported as a cause of IgE-mediated food allergic reaction and anaphylaxis [9-12]. One suspected risk factor for this allergy has been the ownership of household pet birds, *ie* the handling of birdseed containing sunflower seeds [9].

Although sunflower oil rarely may cause allergic reactions, it is more commonly tolerated due to extremely low protein content after processing [13].

Sunflower “butter” is a product made with roasted sunflower seeds and additional ingredients such as sugars, salts and oils. Large-scale production began after joint efforts between the Agricultural Research Service of the United States Department of Agriculture and a private sunflower seed processor at the beginning of the 21^st^ century, with efforts focused on making the spread palatable [14]. The product, known as SunButter, was unveiled in 2002, but became more widely carried by large retailers in 2011 [15].

The first report of allergic reaction to sunflower seed butter described a confirmed IgE mediated reaction to similar product in Australia [16]. The 5 year old child in this report had egg and peanut allergies, asthma, atopic dermatitis and allergic rhinitis and was introduced to sunflower butter as an alternative protein source. It was speculated that lesser exposures to sunflower seed, via muesli or granola bars, may have provided the route of sensitization.

### Mechanism of sensitization to food allergens

It is postulated that food processing and introduction of food allergens through an impaired skin barrier play an important role in the development of food allergies. Research has suggested that roasting of peanut and emulsification into a “butter” vehicle may play a role in the documented increase in peanut allergy [17,18]. It has also been suggested that exposure to topical creams containing peanut proteins through an impaired skin barrier may result in IgE mediated peanut allergy, while early oral exposure is able to promote tolerance [17]. The dry-roasting of peanut - a common practice in Western cultures but not Eastern ones - has been demonstrated to augment peanut’s immunogenicity, with cutaneous sensitization provoking further hypersensitivity in the gut on subsequent oral antigen exposure in a mouse model [19]. Consistent with these theories, household peanut consumption is correlated with risk of peanut allergy in children [20].

## Conclusions

A case of sunflower allergy in the context of household consumption of sunflower butter has not yet been reported. We suggest that consumption of sunflower seed and possibly other “butters”, as peanut alternatives, may lead to sensitization with less common allergens in those with impaired skin barrier, if cutaneous exposure precedes oral introduction and development of tolerance. Homes that are intentionally peanut-safe may provide the environment whereby infants with impaired skin barrier are at increased risk when alternative “butter” products are being used. By virtue of roasting and emulsification, the allergenicity of sunflower seeds may be optimized in the creation of a “butter” product, but further research is required in this regard.

## Consent

Written informed consent was obtained from the patient’s parent for publication of this case report. A copy of the written consent is available for review by the Editor-in-Chief of this journal.
